# IL-1 Receptor Antagonist Blocks the Lipopolysaccharide-Induced Inhibition of Gastric Motility in Freely Moving Conscious Rats

**DOI:** 10.1007/s10620-012-2210-8

**Published:** 2012-05-19

**Authors:** Yoshihiro Tsuchiya, Tsukasa Nozu, Shima Kumei, Masumi Ohhira, Toshikatsu Okumura

**Affiliations:** 1 Department of General Medicine, Asahikawa Medical University, Midorigaoka Higashi 2-1-1, Asahikawa, 078-8510 Japan; 2 Department of Regional Medicine and Education, Asahikawa Medical University, Midorigaoka Higashi 2-1-1, Asahikawa, 078-8510 Japan

**Keywords:** Lipopolysaccharide, IL-1, IL-1 receptor antagonist, Gastric motility, Rat

## Abstract

**Background and Aims:**

Endotoxin/lipopolysaccharide (LPS) alters gastrointestinal functions. However, little is known as to whether LPS could change gastric antral contractility in freely moving conscious animals. We tried to clarify this problem and the associated mechanisms.

**Methods:**

In this study, we recorded intraluminal gastric pressure waves in freely moving conscious rats by manometric catheter located in the antrum. Area under the manometric trace was evaluated as motor index (MI).

**Results:**

Intraperitoneal injection of LPS at doses of 0.2 mg/kg or more significantly inhibited MI. The inhibition started immediately after the administration of LPS and lasted over 1 h. Intraperitoneal injection of IL-1β potently decreased MI while neither IL-6 nor TNF-α inhibited gastric motility, suggesting IL-1β specifically reduced gastric motility. Next, we examined the hypothesis that endogenous IL-1 mediates the LPS-induced inhibition of gastric motility. To address the speculation, an IL-1 receptor antagonist (IL-1Ra) was used to block IL-1 signaling. Pretreatment with IL-1Ra at a dose of 20 mg/kg significantly blocked the inhibition of gastric contractility by LPS at a dose of 0.2 mg/kg.

**Conclusions:**

These results suggest for the first time that LPS or IL-1β is capable of inhibiting gastric motility in conscious rats and that endogenously released IL-1 may mediate the LPS-evoked inhibition of gastric antral motility. This evidence also led us to speculate that IL-1Ra may be a therapeutic tool for patients with disturbed gastrointestinal functions under septic conditions.

## Introduction

Sepsis induces the disturbance of gastrointestinal motility [1]. A therapeutic approach to improve gastrointestinal motility could break the intestinal cycle of sepsis and prevent the development of a sepsis-induced systemic inflammatory response syndrome and multiple organ failure [1]. Regrettably, very limited knowledge exists regarding the pathogenesis of sepsis-induced gastrointestinal motility disturbance [2, 3], and it remains to be fully elucidated whether there are mechanistically microbial-mediated alterations in gut motility. Exogenous lipopolysaccharide (LPS) from gram-negative bacteria is known to be a causative factor of sepsis-induced pathophysiological changes [4].

A number of studies have examined the effect of endotoxin/LPS on gastrointestinal functions. For example, Pons et al. [5] have demonstrated that intestinal myoelectric activity was inhibited by intravenous administration of LPS at a dose of 0.05 mg/kg in conscious fasted rats chronically implanted with nichrome electrodes in the duodenojejunum. With regard to the effect of LPS on gastric functions, gastric acid secretion and gastric emptying were inhibited by systemic administration of LPS in conscious animals [6–11]. A couple of studies have examined the effect of LPS on gastric contractility. In anesthetized rats, Quintana et al. [12] have demonstrated that intravenous injection of LPS at a dose of 0.04 mg/kg by itself failed to change gastric pressure but could inhibit the stimulated gastric tone induced by 2-deoxy-d-glucose in urethane-anesthetized rats. Watanabe et al. [13] measured gastric motility by force transducers in conscious fasted rats and reported that within 2–3 min after LPS at a dose of 1 mg/kg injected intravenously, strong repetitive contraction waves emerged and lasted for 15–20 min. After that, motility was suppressed for more than 2 h. It was also reported that a 0.3 mg/kg dose of LPS failed to inhibit gastric contractility. Thus the dose–response effects of LPS, and the effects of LPS on gastric motility, i.e., inhibition or stimulation, remain controversial. We therefore tried to clarify the effects of LPS on gastric contractility and its mechanisms in freely moving conscious fed rats in this study.

## Materials and Methods

### Chemicals

LPS used in the present study was obtained from *Escherichia* *coli* with the serotype 055:B5 (Sigma Chemical, USA). IL-1β, TNF-α and IL-6 were purchased from Wako Pure Chemical Industries, Ltd, Japan. Recombinant human IL-1 receptor antagonist (IL-1Ra) (anakinra) was purchased from Biovitrum, Sweden. These chemicals were dissolved in endotoxin-free physiological saline before injected.

#### Animal Preparation

Male Sprague–Dawley rats weighing approximately 250 g were housed under controlled light/dark conditions with the room temperature regulated at 23–25 °C. Rats had free access to rat chow (solid rat chow, Oriental Yeast Co., Tokyo, Japan) and tap water.

Intragastric pressure was detected by manometric methods as described in recent publications [14, 15]. Under ether anesthesia, an open-tipped catheter (3-Fr, 1 mm ID; Atom, Tokyo, Japan) was inserted into the gastric antrum to measure gastric tone. The catheter was fixed by sutures at the place of exit from the gastric wall, taken out together through the abdominal wall, and tunneled through the subcutaneous to exit at the skin at the back neck. Rats were maintained in individual cages for approximately 3 days before the experiments.

#### Measurement of Gastric Motility and Experimental Protocols

Conscious fed rats were placed in wire-bottom and non restraint polycarbonate cages. To avoid biting and allow free movement, the manometric catheter was passed through a flexible metal sheath and connected to an infusion swivel (Instech Laboratories, Plymouth Meeting, USA). The end of the catheter was then connected to a pressure transducer (TP-400T; Nihon Koden Kogyo, Tokyo, Japan). Degassed distilled water was continuously infused at a rate of 1.5 ml/h by a heavy-duty pump (CVF-3100; Nihon Koden Kogyo). Gastric pressures were measured and recorded in a Power Lab system (AD Instruments, Colorado Springs, USA). One hour of stabilization, and measurement of gastric pressure waves was initiated. Then, the gastric pressure at the basal state was measured and recorded for 1 h. In the next step, the rats were removed from their cages to receive intraperitoneal injections. Rats were anesthetized with ether and received intraperitoneal injection of each chemical (0.3 ml). After the administration, rats were returned to the cages again and the catheter was re-connected to the recording system, and the intragastric pressure was recorded for 2 h. In a separate set of experiments, recombinant human IL-1Ra at a dose of 20 mg/kg (0.3 ml) or saline (0.3 ml) was injected intraperitoneally under brief ether anesthesia 30 min before intraperitoneal injection of LPS at a dose of 0.2 mg/kg.

#### Calculating the Motor Index (MI)

The motor index (MI) was evaluated by area under the manometric trace (AUT), which was calculated using the software LabChart v7 (AD instruments, Colorado Springs, USA). First, MI at the basal state was calculated. Next, the %MI was determined by calculating as follows: (AUT for each 1 h period after each intraperitoneal injection of tested chemicals)/(basal MI) × 100. In the present experiments, gastric pressure waves were continuously recorded up to 4 h (2 h for stabilization [1 h] and basal MI [1 h] and 2 h after tested chemicals). During the process, measurement was temporally interrupted to inject intraperitoneally tested solutions. To get the appropriate recordings for the analysis, the data during the recovery period for 5 min was excluded from later analysis.

### Statistical Analysis

For statistical analysis of the data, data were expressed as means ± SE. Student’s *t* test was used for comparison of %MI between vehicle + LPS and IL-1Ra + LPS injection. The other data were analyzed by one-way ANOVA followed by Dunnett’s multiple comparison test. Values of *P* < 0.05 were considered statistically significant.

### Ethical Considerations

The approval of the Research and Development and Animal Care committees at the Asahikawa Medical University was obtained for all studies.

## Results

**Fig. 1:**
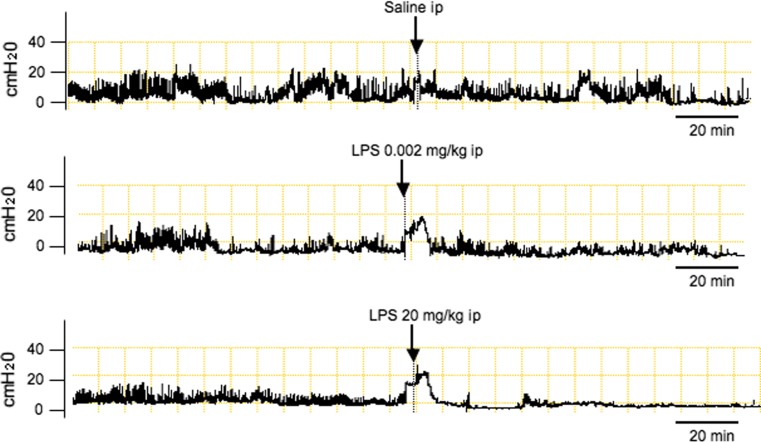
Representative recordings of gastric antral contractions in rats injected intraperitoneally with either saline, 0.002 or 20 mg/kg of lipopolysaccharide (LPS)

First, we examined the effects of intraperitoneal injection of LPS on gastric antral motility in freely moving conscious rats. As demonstrated in Fig. 1, saline administration did not affect the intragastric pressure. LPS at a dose of 20 mg/kg potently suppressed gastric antral contractility while a 0.002 mg/kg dose of LPS failed to inhibit gastric motility. The inhibitory action of LPS was observed immediately after intraperitoneal injection of LPS and persisted more than 60 min. As demonstrated in Table 1, a significant inhibition of MI was observed when LPS was injected at doses of 0.2 mg/kg or more.

**Table 1:** Effects of intraperitoneal injection of chemicals on gastric antral motility in conscious rats

Treatment	*N*	%MI
Saline	5	90.9 ± 5.4
LPS (mg/kg)		
0.002	4	100.3 ± 10.1
0.2	5	44.3 ± 6.5^a^
20	7	49.3 ± 7.4^a^
IL-1β (μg/kg)		
0.2	6	76.2 ± 5.4
2	5	33.8 ± 6.6^a^
TNF-α (μg/kg)		
2	6	101.5 ± 14.9
20	7	80.9 ± 5.4
IL-6 (μg/kg)		
0.2	4	79.4 ± 6.4
2	7	81.6 ± 12.2
IL-1Ra (mg/kg)		
20	5	94.3 ± 15.9
Saline + LPS (0.2 mg/kg)	7	44.2 ± 9.2
IL-1Ra (20 mg/kg) + LPS (0.2 mg/kg)	5	96.0 ± 8.8^b^

Each data represents the mean ± SE

^a^ *P* < 0.05, when compared with saline

^b^ *P* < 0.05, when compared with saline + LPS (0.2 mg/kg)

**Fig. 2:**
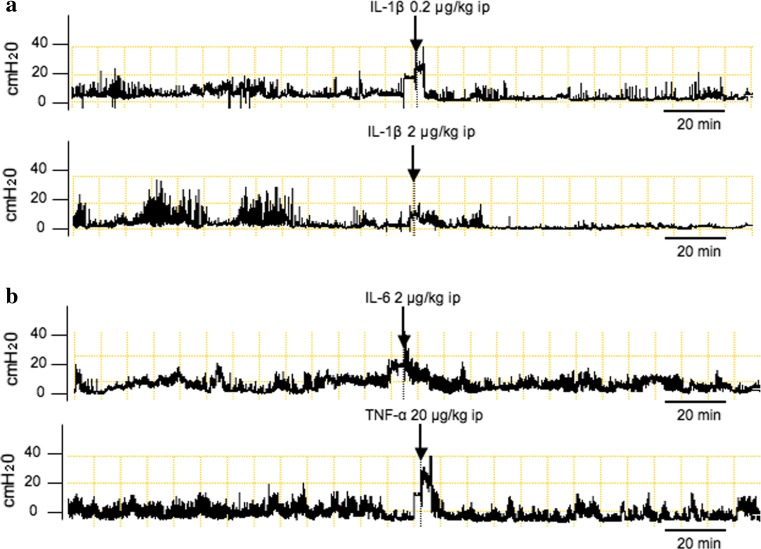
Representative recordings of gastric antral contractions in rats injected intraperitoneally with either IL-1β (**a**), TNF-α or IL-6 (**b**)

To evaluate the mechanisms by which LPS inhibit gastric motility, effects of cytokines such as IL-1β, -6 or TNF-α, which are possibly induced by LPS on gastric motility, were examined. Figure 2a reveals that intraperitoneal injection of IL-1β at a dose of 2 μg/kg strongly suppressed gastric antral motility. The inhibition was observed for more than 60 min. As demonstrated in Table 1, intraperitoneal injection of IL-1β decreased MI in a dose dependent manner. On the other hand, TNF-α at doses of 2 and 20 μg/kg or IL-6 at doses of 0.2 and 2 μg/kg failed to change gastric motility as shown in Fig. 2b and Table 1, suggesting that among tested cytokines, IL-1β specifically inhibited gastric motility.

**Fig. 3:**
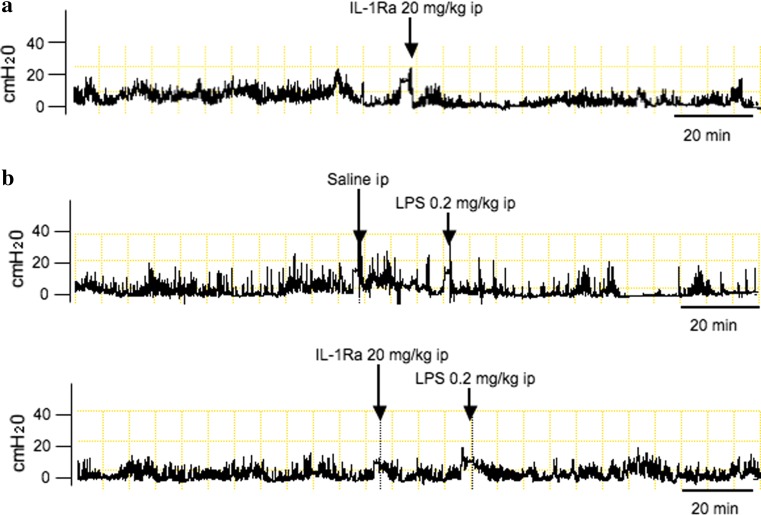
Representative recordings of gastric antral contractions in rats injected with IL-1Ra alone (**a**) or either saline or IL-1Ra plus intraperitoneal injection of LPS at a dose of 0.2 mg/kg (**b**)

Next, to clarify the hypothesis that endogenously released IL-1 mediates the LPS-induced inhibition of gastric motility, we examined the effects of IL-1Ra on the LPS-induced suppression of gastric motility. Intraperitoneal injection of IL-1Ra at a dose of 20 mg/kg by itself did not change gastric motility (Fig. 3a; Table 1). In contrast, pretreatment with IL-1Ra significantly blocked the inhibition of gastric motility by intraperitoneal LPS at a dose of 0.2 mg/kg as shown in Fig. 3b and Table 1, suggesting that endogenously released IL-1 could be involved in the inhibition of gastric motility by LPS.

## Discussion

The present study clearly demonstrated that LPS at doses of 0.2 mg/kg and more suppressed gastric antral motility in freely moving conscious rats. Earlier investigators demonstrated that intravenous injection of LPS at a dose of 0.04 mg/kg by itself failed to change gastric pressure but could inhibit the stimulated gastric tone induced by 2-deoxy-d-glucose in urethane-anesthetized rats [12]. In contrast, Watanabe et al. [13] have shown that gastric motility detected by force transducers in conscious fasted rats was stimulated within 2–3 min after intravenous injection of LPS at a dose of 1 mg/kg injected intravenously, and repetitive contraction waves emerged and continued for 15–20 min, and then motility was suppressed for more than 2 h. It was also reported in the study that a 0.3 mg/kg dose of LPS failed to inhibit gastric contractility. The discrepancy of the effect of LPS on gastric motility between the present study and the previous studies as described above might come from the difference of fasted or fed state, anesthetized or conscious, and/or force transducers or manometric method. As reported by previous researchers, LPS potently inhibits gastric emptying. Since gastric antral motility is deeply involved in the gastric emptying [16], the present data may suggest that inhibition of gastric antral motility by LPS in conscious rats contributes to the delayed gastric emptying seen in previous studies.

LPS causes the release by the host of numerous proinflammatory mediators, such as TNF-α, IL-6 and IL-1β through activation of NF-κB [17]. To clarify the mechanism of how LPS suppresses gastric motility in conscious rats, we examined the effects of LPS-induced cytokines, TNF-α, IL-6 or IL-1β on gastric antral contractility. The present study clearly demonstrated that IL-1β dose-dependently suppressed gastric antral motility while neither IL-6 nor TNF-α inhibited gastric motility in freely moving conscious fed rats. These results suggest that among inflammatory cytokines, IL-1β is specifically capable of inhibiting gastric motility.

A couple of reports demonstrated that TNF-α acts in the brain to suppress gastric tone in anesthetized rats. For instance, TNF-α injected directly into the dorsal motor nucleus (DMN) in the medulla oblangata, the cells of origin innervating the stomach through the vagus nerve [18], causes a reduction of gastric tone [19]. In addition, continuous perfusion of the floor of the fourth ventricle with TNF absorbant construct reversed the inhibition of gastric motility by LPS [20]. Based upon this evidence, TNF-α may act in the brain to be involved in the inhibition of gastric function by LPS. The present study, however, showed that intraperitoneal injection of TNF-α failed to change gastric tone, suggesting that gastric motility could not be changed by circulating TNF-α. These results led us to speculate that direct application of TNF-α into the brain might be needed to change gastric motility. Otherwise, the discrepancy between the evidence by earlier investigators and our results in this study may come from the difference of anesthetized or conscious rats. Further studies should be performed to clarify the issue.

Most previous studies of the effect of IL-1β on gastrointestinal motility have concerned the small and large intestines. For example, Fargeas et al. [21] revealed that centrally administered IL-1β at a dose of 15 ng potently stimulated cecocolonic motility in conscious rats. On the other hand, peripheral injection of IL-1β failed to stimulate cecocolonic motility, suggesting that IL-1β acts centrally in the brain to stimulate lower GI motility. With regard to the effect of IL-1β on gastric motility, a couple of studies have shown that IL-1β modifies gastric motility in experimental animals. Montuschi et al. [22] demonstrated that IL-1β is capable of inducing dose-dependent relaxation in rat gastric fundus strip in vitro, suggesting that IL-1β acts directly in the stomach to relax gastric smooth muscle. According to the report by Sütö et al. [23], IL-1β injected intracisternally or intravenously dose-dependently decreased gastric emptying, respectively. The median effective dose (ED50) was 30-fold lower when IL-1β was injected intracisternally rather than intravenously, suggesting a site of action of IL-1β should be in the brain. Thus, it has not been established whether the site of action of IL-1β is on the brain and/or the stomach. In addition, we could not find any recordings of gastric contractility in conscious animals injected with IL-1β. The present study therefore clearly demonstrated for the first time that IL-1β potently inhibits gastric contractility in freely moving conscious rats. Further studies should be needed to clarify if IL-1β acts in the brain to inhibit gastric tone.

The present study demonstrated that each LPS or IL-1β strongly inhibited gastric motility in conscious rats. Based upon the findings, we made a hypothesis that IL-1β mediates the LPS-induced inhibition of gastric motility. In the next step, we tried to clarify the hypothesis using an IL-1Ra.

The action of IL-1 is mediated by two different gene products, IL-1α and IL-1β [24, 25]. These two cytokines bind to IL-1 receptor-1 (IL-1R1). The effects of IL-1 are strictly regulated by naturally occurring inhibitors, such as IL-1Ra [26]. It has been established that IL-1Ra binds to IL-1R1 and thus antagonizes biological effects of IL-1 [27]. Anakinra used in this study is a nonglycosylated, recombinant form of human IL-1Ra that, like endogenous IL-1Ra, competitively inhibits IL-1 by binding IL-1 R1 and regulates negatively the IL-1 signaling [28, 29]. In experimental animals, IL-1Ra is effective to antagonize the influence of IL-1 β [30, 31]. Terrando et al. [30] have shown that administration of IL-1Ra reduced hippocampal microgliosis and ameliorated cognitive dysfunction induced by intraperitoneal injection of LPS in mice, suggesting that IL-1 signaling by LPS may induce microglial activation in the brain and lead to behavioral abnormality. Auvin et al. [32] demonstrated that inflammation provoked by LPS enhanced rapid kindling epileptogenesis in immature rat brains. In this model, IL-1Ra was able to mitigate the augmentation of epileptogenesis enhance by LPS, suggesting IL-1 signaling may partly mediate the epileptogenesis by LPS. In the experiments, IL-1Ra at a dose of 25 mg/kg was administered intraperitoneally to block the effect of intraperitoneal injection of LPS. Girard et al. [31] reported that IL-1Ra protected against placental and neurodevelopmental defects induced by LPS-induced maternal inflammatory response in rats. In the study, IL-1Ra at doses of 2–20 mg/kg was used to block the LPS (0.2 mg/kg)-induced effect in rats. The dose of IL-1Ra (20 mg/kg) used in the present study was therefore selected according to the above reports.

As demonstrated in this study, IL-1Ra drastically blocked the LPS-induced inhibition of gastric motility while IL-1Ra by itself failed to change gastric motility, suggesting for the first time that IL-1 mediates the LPS-induced inhibition of gastric motility. Because little is known whether endogenously released IL-1 is indeed implicated in the gastrointestinal functions, the present study provides a novel evidence that endogenously released IL-1 indeed plays a key role in the regulation of gastrointestinal functions.

IL-1Ra has been tested clinically in patients with rheumatoid arthritis, with small effect [33]. On the other hand, beneficial effects by IL-1Ra were observed in patients with cryopyrinopathie, which is known as autoinflammatory conditions closely associated with excessive IL-1 signaling [26]. IL-1Ra has also been reported to be effective in Schnizler syndrome, systemic-onset juvenile idiopathic arthritis and adult Still disease [26]. Furthermore, recent studies have demonstrated that IL-1 targeting is efficacious in type 2 diabetes [34] and gout [35]. In addition to the previous findings, the present evidence that IL-1Ra blocks the LPS-induced change of gastric motility in conscious rats may contribute to develop a novel therapeutic approach to improve gastrointestinal motility disturbance under septic conditions.

## Abbreviations

LPS: Lipopolysaccharide
IL-1Ra: IL-1 receptor antagonist
